# Phylogeographic Triangulation: Using Predator-Prey-Parasite Interactions to Infer Population History from Partial Genetic Information

**DOI:** 10.1371/journal.pone.0050877

**Published:** 2012-11-28

**Authors:** A. Márcia Barbosa, Guillermo Thode, Raimundo Real, Carlos Feliu, J. Mario Vargas

**Affiliations:** 1 Cátedra ‘Rui Nabeiro’ – Biodiversidade, Centro de Investigação em Biodiversidade e Recursos Genéticos (CIBIO), Universidade de Évora, Évora, Portugal; 2 Division of Biology, Imperial College London, Ascot, United Kingdom; 3 Departamento de Biología Celular y Genética, Universidad de Málaga, Málaga, Spain; 4 Laboratorio de Biogeografía, Diversidad y Conservación, Departamento de Biología Animal, Universidad de Málaga, Málaga, Spain; 5 Unitat de Parasitologia, Departament de Microbiologia i Parasitologia Sanitàries, Universitat de Barcelona, Barcelona, Spain; Washington State University, United States of America

## Abstract

Phylogeographic studies, which infer population history and dispersal movements from intra-specific spatial genetic variation, require expensive and time-consuming analyses that are not always feasible, especially in the case of rare or endangered species. On the other hand, comparative phylogeography of species involved in close biotic interactions may show congruent patterns depending on the specificity of the relationship. Consequently, the phylogeography of a parasite that needs two hosts to complete its life cycle should reflect population history traits of both hosts. Population movements evidenced by the parasite’s phylogeography that are not reflected in the phylogeography of one of these hosts may thus be attributed to the other host. Using the wild rabbit (*Oryctolagus cuniculus*) and a parasitic tapeworm (*Taenia pisiformis*) as an example, we propose comparing the phylogeography of easily available organisms such as game species and their specific heteroxenous parasites to infer population movements of definitive host/predator species, independently of performing genetic analyses on the latter. This may be an interesting approach for indirectly studying the history of species whose phylogeography is difficult to analyse directly.

## Introduction

Phylogeography examines the geographical distribution of intra-specific genetic lineages to infer historical information such as the location of glacial refuges and subsequent migratory routes. It requires gathering geographically dispersed biological samples and analysing rapidly-evolving genetic markers such as mitochondrial genes [1]. Phylogeographic studies are expensive, time-consuming, and barely practicable for scarce species for which an adequate number of DNA samples is difficult to obtain. Non-invasive methods exist for those cases [2], but they are more expensive and less efficient.

Co-structure analysis has been defined as the comparison of population demographic and/or genetic structures between two or more species, with the aim of elucidating factors that determine that structure in one or more of those species [3]. Comparative phylogeography of ecologically related species can thus provide a valuable insight into the role of historical factors in their observed distribution patterns. The phylogeographies of species linked by a close biotic interaction such as parasitism show a degree of congruence that tends to increase with the obligate character of the parasite [4]. Moreover, as parasites can have a higher molecular evolution rate, the phylogeography of a specific parasite may work as a “biological magnifier” over the phylogeography of its host, revealing cryptic aspects of the history of its populations [5]. The concordance decreases if the parasite is either not specific or heteroxenous, i.e., uses more than one host species to complete its lifecycle [3], [6]–[8].

Predator-prey interactions may also, by a similar logic, contribute to generating congruence in species’ population histories. Moreover, the phylogeography of a parasite with a prey species as intermediate host and its predator as definitive host should reflect population history traits of both the prey and the predator hosts. If the parasite has no additional hosts and no free dispersing lifecycle phases, any migratory movement indicated by the parasite’s phylogeography that is not reflected in one of the hosts can be attributed to population movements of the other host [3], [7], [9]. It may thus be possible to use the phylogeography of, for example, a game species and a specific heteroxenous parasite, with several high-quality biological samples easily available from hunters, to infer population history traits of its predators without the need for direct genetic analyses on the latter.

**Figure 1:**
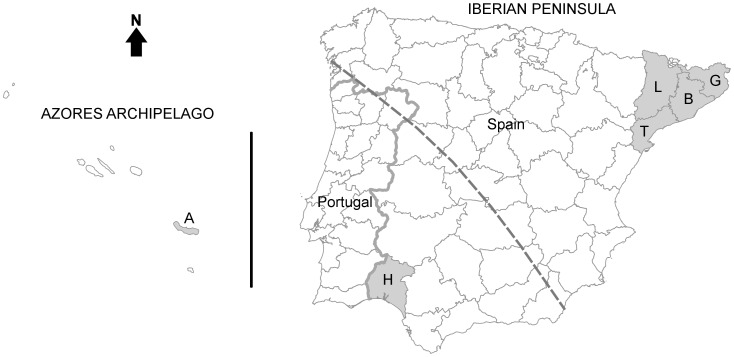
Geographic distribution of the analysed *Taenia pisiformis* individuals. Iberian Peninsula, the Azores archipelago and (shaded in grey) the regions of origin of the analysed sequences. Province initials correspond with those in Table 1. The dashed line is the putative limit between the north-eastern and south-western Iberian rabbit (intermediate host) lineages [13].

We used, as an exploratory example, samples of a tapeworm (*Taenia pisiformis*) that were stored in a parasitological collection [10] including individuals from two parts of the Iberian Peninsula (SW Europe) and from the Archipelago of the Azores (N Atlantic Ocean; Figure 1). This tapeworm uses rabbits (*Oryctolagus cuniculus*; very rarely hares *Lepus* spp.) as intermediate hosts and canids and felids as definitive hosts. Several studies on rabbit phylogeography have revealed the existence of two clearly divergent and practically allopatric lineages: one in the south-western half of the Iberian Peninsula and in the Azorean islands, and the other in the remaining rabbit distribution range, including the north-eastern Iberian Peninsula, the remaining European countries, and Australia [11]–[14] (Figure 1). We analysed phylogeographic traits of the tapeworm and compared them to the known phylogeography of the rabbit. Our central aim was to demonstrate the use of comparative phylogeography within predator-prey-parasite relationships to uncover dispersal movements of host species whose phylogeography may be difficult to analyze directly. We detected incongruences between the rabbit and the tapeworm’s spatial genetic structures that may be attributed to migratory movements of predators of the rabbit that act as definitive hosts for this parasite.

**Table 1:** Variation in *Taenia pisiformis CO*I sequences.

Code	Locality andprovince of origin	Region	Haplotype
B1	El Papiol, Barcelona	NE	CCCCGCAAAATTACTTAA
A1	São Miguel, Açores	Az	………………
A2	São Miguel, Açores	Az	………………
A3	São Miguel, Açores	Az	………………
A4	São Miguel, Açores	Az	………………
B8	Collsuspina, Barcelona	NE	….A………….
G1	Vilobí d'Onyar, Girona	NE	….A………….
L3	Tàrrega, Lleida	NE	….A………….
L4	Sant Pere dels Arquells, Lleida	NE	….A………….
T1	Valls, Tarragona	NE	….A………….
H4	Matas Gordas, Huelva	SW	….A………….
H5	Matas Gordas, Huelva	SW	….A………….
B10	Centelles, Barcelona	NE	.T.A………….
B7	La Guardia – El Bruc, Barcelona	NE	….A….C…….
B6	Les Franqueses del Vallès,Barcelona	NE	….A………C.G
H2	Puebla de Guzmán, Huelva	SW	.T.A………C…
B2	Sitges, Barcelona	NE	.T.TA…G………
H3	Matas Gordas, Huelva	SW	.T.A.G……T….
L2	Sant Pere dels Arquells, Lleida	NE	T…A……C.TC…
B3	Caldes de Montbui, Barcelona	NE	….A……CGTCC.
T2	Valls, Tarragona	NE	….AT.G.G.C.TC.G.

Individuals of *T. pisiformis* whose *CO*I was sequenced, their geographic origin (province code letters correspond with those in Figure 1) and haplotype. Only variable positions are shown; complete sequences are published in GenBank (accession numbers KC020690 to KC020710). Dots represent consensus with the first sequence in the list. NE: North-Eastern Iberian Peninsula. SW: South-Western Iberian Peninsula; Az: Azores.

## Materials and Methods

DNA was salt-extracted [15] from 17 *T. pisiformis* individuals from NE Iberian Peninsula, 9 from SW Iberian Peninsula, and 4 from the Azores (Figure 1). Each individual had been recovered from a separate rabbit host (shot by licensed hunters during the game season) and preserved in 70% ethanol for several years [10]. DNA was thus scarce and degraded, so we chose a relatively small mitochondrial DNA sequence to analyze, the cytochrome oxidase C subunit I (*CO*I), which had already been analysed successfully in other flatworm (Platyhelminthes: Cestoda) species [16], [17].

A first degenerate primer pair (*Taenia*_COI-F: 5′-TGG TCW GGT TTT GTR GGT TTA AG and *Taenia*_COI-R: 5′-GCM ACM ACA AAY CAA GTA TC) flanking 1100 bp was designed from relatively conserved marginal regions of the *CO*I gene of *T. asiatica*, *T. solium* and *T. crassiceps*, whose mitochondrial DNA sequences were available in GenBank (accession numbers NC 004826, NC 004022, and NC 002547, respectively). These primers achieved amplification in 8 of our *T. pisiformis* samples, whose sequences were then aligned to design a second nested pair of primers (*Tp*_COI-F: 5′-CTA ATC ACG GTA TAA TCA TG-3′ and *Tp*_COI-R: 5′-CCA GTT ACA CCT CCA AAA G-3′) flanking 885 bp for amplifying the remaining samples.

Polymerase Chain Reactions (PCR) were carried out on 20-µL volumes containing 0.88 µL dNTPs (10 mmol/L), 0.8 µL each primer (10 µmol/L), 0.2 µL EcoTaq polymerase (5 units/mL), 2 µL DNA sample, 2 µL reaction buffer, and 0.8 to 1.6 µL MgCl_2_ (successive adjustments in MgCl_2_ concentration were necessary to amplify all sequences). Reactions were cycled 30 times as follows: 30 sec at 94°C, 30 sec at 50°C, and 30 sec at 72°C, with an initial denaturation of 5 min at 94°C and a final elongation of 7 min at 72°C. A total of 21 samples were amplified successfully (Table 1). PCR products were purified using ExoSAP-IT (GE Healthcare) according to the manufacturer’s instructions, and then sequenced on both strands by an independent laboratory (STAB vida, Oeiras, Portugal) using the PCR primers. Sequences were aligned with BioEdit [18]. A total of 777 bp could be compared among all samples.

We used the *ape* package [19] in R 2.11 [20] to quantify the genetic dissimilarity [21] between the analysed sequences. The limited sample size prevented obtaining robust clusters for a reliable phylogeographic tree; instead, we carried out multidimensional scaling, also known as principal coordinates analysis [22], to obtain a two-dimensional representation of the genetic distances between these sequences. This allowed visual comparison with their geographic distances, which were mapped with Quantum GIS 1.8 Lisboa [23].

## Results and Discussion

We found 18 variable positions (18 mutations) forming 11 different haplotypes, most of which in the individuals from the north-eastern Iberian Peninsula (Tables 1 and 2). This may be due to the greater number of individuals analysed in the north-eastern region (Table 2), although it might also be related to a greater abundance in this area of this parasite’s wild hosts, rabbit and red fox (*Vulpes vulpes*), as suggested by environmental favourability models for these species [24]–[26]. Such models have shown to correlate with abundance data where these were available [25], [26]. Host abundance is often directly related to parasite abundance and, hence, parasite diversity [24], [27]. However, haplotype diversity, which accounts for the number of analysed individuals in each region, was only slightly higher in the north-eastern than in the south-western Iberian Peninsula (Table 2). In rabbits, genetic diversity is clearly higher in south-western than in north-eastern Iberian populations [13], [14].

**Table 2:** Genetic diversity of *Taenia pisiformis* populations.

	Individuals	Haplotypes	Mutations	Hd
Northeast	13	9	16	0.87
Southwest	4	3	5	0.83
Azores	4	1	0	0.00
TOTAL	21	11	18	0.85

Number of individuals, haplotypes, and mutations encountered, and haplotype diversity (Hd) for each region studied and for the whole study area.

**Figure 2:**
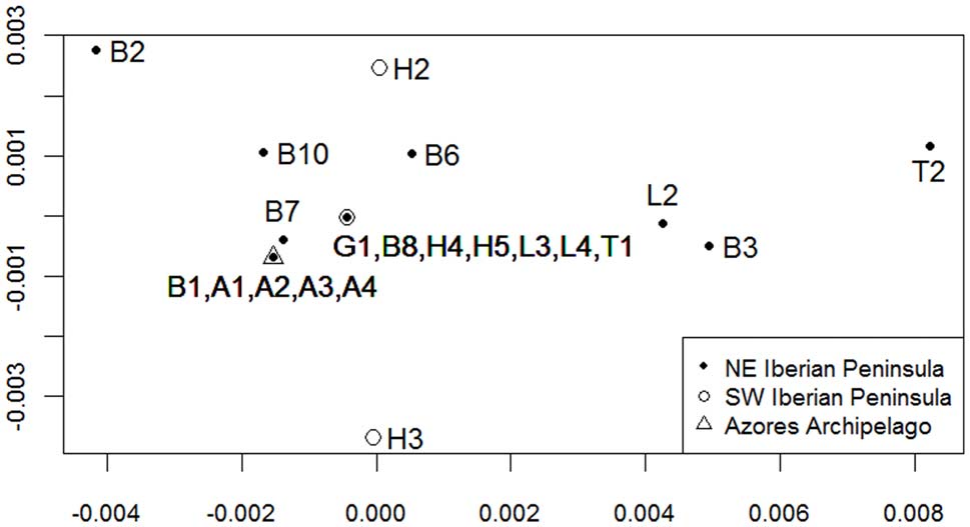
Multidimensional scaling of the genetic distances between the analysed individuals. Values in the axes are coordinates generated to provide a two-dimensional representation of the genetic dissimilarity between the *Taenia pisiformis CO*I sequences (individual codes correspond with those in Table 1, province initials with Figure 1). The symbols represent their main regions of origin.

Despite some genetic divergence, such as that of individual H3, most tapeworm individuals from south-western Iberia were closely similar to others from north-eastern Iberia (Table 1, Figure 2). These genetic similarities among north-eastern and south-western individuals indicate the absence of a clear geographic differentiation among the Iberian populations of this parasite, despite the clear spatial genetic structure that has been observed its intermediate host, the wild rabbit [12]–[14]. The existence of definitive host species with greater mobility, such as foxes, dogs and cats, may explain the lack of a clear genetic structure in this tapeworm at the analysed scale. Indeed, previous studies on the phylogeography of the red fox indicate relatively uniform, not spatially structured genetic patterns in our study area [28], [29]. Additionally, domestic dogs and cats are frequently transported by humans. The presence of four tapeworm individuals in the Azores with the same haplotype as the north-eastern B1 individual (Table 1, Figure 2) also suggests recent transportation by a definitive host (in this case, most probably a domestic animal) rather than by a wild rabbit, as Azorean rabbits are genetically closer to those from western Iberia [14]. Human intervention is known to affect many species’ phylogeographic patterns [8].

The particular results presented here have a mainly exploratory interest, given the reduced sample size, host specificity, geographical coverage and genetic marker set analysed. Our study was based on biological samples that were available in a parasitological collection. Biological collections are fundamental resources for studies concerning species and biodiversity trends, biological invasions, public health and safety, and many other important areas [30]. Some changes to procedures associated with biological collections could mitigate some of their biases and limitations, making such collections more useful [31]. Host-parasite comparative studies can be a valuable tool for a number of purposes and, ideally, they should be based on directly comparable individuals – that is, on genetic sequences of parasites and hosts sampled simultaneously. It would thus be useful to include in parasitological collections a biological sample of the host individual containing each parasite pool, as a standard procedure to make future co-structure or co-phylogeography analyses more feasible, rigorous and valuable.

Some parasite traits, such as effective population size, generation time, mutation rate and level of host specificity, may be important for allowing accurate inferences on host history. Ideally, there should be a match between these traits and the timescales (phylogenetic, phylogeographic and demographic) that are relevant to the issues at hand [32]. Different rates of evolution between hosts and parasites can make it difficult to synchronize observed molecular changes in time [8]. In spite of the limitations outlined above, the results presented here unequivocally show that comparative phylogeography of a parasite and its intermediate prey host can provide evidence for population movements of a definitive predator host, independently of genetic analysis of the latter. Our analysed sample clearly documents recent geographic movements of *T. pisiformis* that were unlikely led by its host rabbits, as rabbit genetic lineages have long been segregated [12]–[14]. They therefore reflect dispersal or migration movements of the predators/definitive hosts, which have effectively remained more mobile [28], [29].

It is well accepted that, if the phylogeographies of a heteroxenous parasite and one of its hosts do not match, the discrepancy can be explained by the dispersal provided by other hosts in the parasite’s life cycle [3], [7], [9]. It has also been suggested that, when a host or geographic region is of conservation concern, parasite data can be used to support the boundaries of historically unique regions or managed host populations [33]; and that genetic assignment of parasites may be useful to identify dispersal patterns or feeding grounds for migratory host species [3], among several other interesting applications [8]. What we propose here is that co-structure or co-phylogeography be used as a tool to specifically detect dispersal movements and other population history traits of difficult-to-analyse species that are involved in predator-prey-parasite triangles. Species whose phylogeography is hard to analyse directly include not only rare and endangered species for which adequate DNA samples are difficult to obtain in sufficient quantity, but also common predator species, which often have large home ranges and high dispersal ability, so their spatial genetic structures tend to be less well-defined and their migratory movements more difficult to detect from their own genetic structure [29]. Our results show that information on the genetic structure of their prey and shared parasites can reveal, via triangulated inference, unnoticed population dynamics traits (such as particular dispersal movements) of predators. This application can be especially valuable given the current conservation crisis, which is compounded in apex predators such as carnivores and raptors.
